# The great American car crime decline

**DOI:** 10.1057/s41284-024-00452-2

**Published:** 2025-01-04

**Authors:** Graham Farrell

**Affiliations:** School of Law, University of Leeds, Leeds, LS2 9JT UK

**Keywords:** Car theft, Engine immobilizer, Crime decline, Crime drop, Security hypothesis, Situational crime prevention, Hot products, Crime concentration, Auto theft

## Abstract

The vehicle theft rate in the United States declined 80 percent between 1990 and 2020. Remarkably, this remains unexplained. This study examines historical evidence including reports to Congress plus automobile industry data from the Federal Register. Legislation incentivised security improvements that were fitted to high-risk vehicles from the late 1980s. Analysis of the industry data finds that theft of vehicles with electronic engine immobilizers declined 80 percent relative to a matched control group and theft of new secure vehicles declined before older vehicles. Theft declined gradually over the years as secure vehicles permeated the national vehicle fleet, the prolonged decline reflecting the arms race between manufacturers’ responses and offender effort to circumvent security. The study concludes that the electronic engine immobilizer caused the great American car crime decline. If this induced declining crime more generally, the electronic engine immobilizer may be the most important crime prevention device of recent history.

## Introduction

Auto theft in the United States rose rapidly in the decades following the Second World War. The *Report of the President’s Commission on Law Enforcement and  the Administration of Justice* attributed a doubling of the car theft rate between 1950 and 1965 to increased post-war ownership and easy opportunities to take a car, recommending that a “more fundamental change in the ignition system and other automobile components is needed.” (President’s Commission, 1967; p. 260). The present study offers new evidence that such a fundamental change caused three decades of decline in theft rates via the introduction and spread of engine immobilizers.

**Fig. 1:**
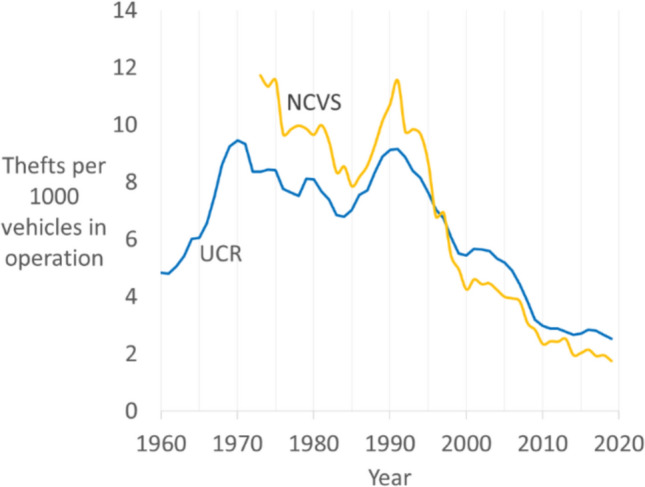
UCR and NCVS vehicle thefts per 1000 vehicles in operation 1960–2019 (Source: UCR; Oakridge National Laboratory)

Figure 1 shows police-recorded Uniform Crime Reports (UCR) trends from 1960, and National Crime Victimization Survey (NCVS) trends from 1973—the first NCVS year—as thefts per 1000 vehicles in operation (Sparks 1981; Clarke and Harris 1992).^1^ Most vehicle theft is reported to the police, so UCR data is a reliable indicator (President’s Commission 1967; p. 22, Harlow 1998; p. 1; Morgan and Truman 2017; Lauritsen et al. 2016). Following the post-war increase, a decline in vehicle theft of around a third from 1970 to the early 1980s (examined further below) was followed by increases to a second peak in 1991. Across the next three decades, NCVS and UCR vehicle theft declined 85 and 72 percent respectively. This major and prolonged decline is important, not only for the understanding and potential prevention of vehicle theft, but for understanding and preventing crime generally due to the prominence of vehicle theft in the ‘great American crime decline’ (Zimring 2007).

 As car theft increased, a 1980’s Bureau of Justice Statistics (BJS) report observed that.

“Motor vehicle theft is of great concern to most Americans … Motor vehicles are the most frequently used form of transportation in the United States. …. Because most people rely on motor vehicles for transportation, when a vehicle is stolen, its theft causes inconvenience to household members, time is often lost from work, and household spending is affected.” (Harlow 1988; p. 1)

And at car theft’s 1991 peak, the Chair of a congressional subcommittee on car theft sounded a rhetorical alarm:

“What epidemic is costing Americans billions of dollars in increased insurance premiums and lost time from work, and claims a new victim every 20 seconds? It’s not a communicable disease, it isn’t cancer but it’s spreading like one. It’s auto kleptomania and it’s sweeping the nation.^2^”

The harmfulness of vehicle theft to victims, their families and society, includes financial and non-financial costs (McCaghy et al. 1977; Clarke and Harris 1992; Miller et al. 1996; Cohen 2000; Wickramasekera et al. 2015). Financial costs include replacement costs of vehicles, repair costs for recovered and damaged vehicles (including from attempts), and The National Highway Traffic Safety Administration (NHTSA 2022) reports that the financial costs of vehicle theft to consumers was $7 billion in 2020, when the theft rate was a fifth that of 1990. Costs include rental of replacement vehicles, of retrieving vehicles from police lots, costs of the inconvenience, and the often considerable psychological and emotional costs of intrusion, trespass and threat to way of life. Other costs include insurance, precautionary measures, costs of theft-related accidents including injury to drivers, passengers, by-standers and others, the economic cost of absences from work, other anti-crime measures as well as policing and criminal justice costs. There are multiplier effects when vehicle theft leads to other crimes including driving offenses, transportation to burglary, robbery or other crime, sale of stolen vehicles and parts, illegal shipments, and use of proceeds including by organized crime. Another multiplier occurs when adolescents, who learn from vehicle theft, progress to other property crimes and violence (White et al. 2008) and when victims incur the longer-term costs of reduced employment and life prospects (Macmillan 2000).

This study is underpinned by crime opportunity theory. A crime opportunity is defined as any situation in which the perceived costs of offending are outweighed by the perceived benefits. This includes the routine activities (Cohen and Felson 1979) and rational choice perspectives (Clarke and Cornish 1985, 1986), and the applied framework of situational crime prevention (Clarke 2012, Eck and Clarke 2018). Given that crime occurs on the convergence of a potential offender with a suitable target without capable guardianship, then the extent and nature of targets and surveillance play a central role in crime. Situational prevention influences offender decisions through modifications to products, systems and the environment that increase the actual or perceived cost, or reduce the actual or perceived benefit, of committing crime. It identifies broad mechanisms by which this occurs—increasing the risk and effort, reducing rewards and provocations, and removing excuses—each linked to more detailed specific mechanisms for inhibiting crime. So, for example, an electronic engine immobilizer (detailed further below) that isolates the ignition and fuel pump is a form of target hardening that works by increasing the effort (including time, skill and resources) required to steal a vehicle, thwarting some offenders and deterring others.

There is considerable evidence that situational crime prevention can reduce crime rates, and that it can be applied to all crime types (Eck and Clarke 2018, Clarke and Bowers 2017). When crime is prevented, offenders often do not displace or may do so partially, while crime can be strategically shifted to less harmful forms (Guerette and Bowers 2009; Barr and Pease 1990). Moreover, a diffusion of preventive benefits can extend impact beyond what was originally intended (Guerette and Bowers 2009), and anticipatory benefits denote how impact can occur before the recognized inception of an intervention (Smith et al. 2002). In the longer-term, offender adaptations of their modus operandi can emerge, in the attempt to circumvent crime prevention interventions, which can prompt further crime prevention developments, resulting in a co-evolutionary arms race (Ekblom 1999).

Crime is always concentrated such that a small proportion of targets, places and victims account for a large proportion of crime (Sherman et al. 1989; Weisburd 2015; Farrell and Pease 2017). A 'hot-products strategy' is a strategic application of situational prevention to one form of crime concentration (Clarke 1999, Bowers and Johnson 2013). Some consumer goods are more likely to be stolen than others because they have desirable characteristics: they are CRAVED: Concealable, Removable, Available, Valuable, Enjoyable, and Disposable (where disposable means easily re-sold). Some product makes and models, such as those of motor vehicles, are disproportionately stolen because they are more attractive. A hot-products strategy is resource efficient and practical because it prioritizes high-risk targets to receive preventive resources.

Installing electronic engine immobilizers (eIMs) on vehicles at high-risk of theft is the application of a hot-products strategy that is central to the present study, and the international research that informs this focus is reviewed next. An eIM is defined by NHTSA as.

“an anti-theft device that combines microchip and transponder technology with engine and fuel immobilizer components that can prevent vehicles from starting unless a verified code is received by the transponder.” (NHTSA 1992).

A more detailed description is that an eIM.

“typically disables two or more electrical circuits (linked to either the ignition and / or fuel pump circuits), built into the engine management system. Although there are various designs, electronic immobilisers most commonly work through a small transponder in the ignition key that transmits a weak radio signal, broadcasting an encrypted code that is picked up by a receiver located close to the ignition lock. When an expected code is received, the electronic immobiliser is disengaged.” (Brown 2015; 330)

### International research

A systematic review of international studies found that “15 of the 16 studies indicate that electronic immobilization has been successful in reducing vehicle theft” that “[t]hese reductions have mostly been larger for temporary (recovered) vehicle thefts than for permanent (unrecovered) thefts,” and that “[a]lthough some studies showed there had been displacement towards vehicles without electronic immobilisation, this was outweighed by the reductions in vehicle theft observed overall.” (Brown 2015; p. 329). That review helped inform what amounts to scientific consensus that eIMs have reduced vehicle crime. However, there is a need to more clearly establish the extent to which eIMs contributed to car theft declines, and that goal is pursued here. Hence, this review section is detailed, because it reassesses existing knowledge.

#### United Kingdom

Studies relating to the UK were earlier and more numerous so are reviewed first. The national car theft rate in England and Wales increased for decades to a peak in 1992, then declined fairly steadily and by 86.4 percent by 2023 (ONS 2024). The ‘reduced pool theory’ attributed the decline to security improvements on new vehicles (Sallybanks and Brown 1998; Brown and Thomas 2003; Laycock 2004; Farrell et al. 2011a, b; Farrell 2015). A series of indicators proved consistent with the expected effects of improved security. Among them, the more prominent included:

- Temporary theft (for joyriding and transportation) was largely eliminated while permanent theft (for resale or parts) was not, consistent with juveniles being easily deterred as theft became more difficult;
- Attempted thefts declined after completed thefts, consistent with some thwarted offenders continuing before quitting;
- Vehicles with an eIM and central door locks were 25 times less likely to be stolen than those without security.

These were argued to be data signatures that, in addition to providing confirmatory evidence, can also rule out alternate explanations (Eck and Madensen 2009). Specifically, other explanations for car crime’s decline are inconsistent with these indicators. There was evidence of crime displacement to older vehicles in the first few years after the introduction of eIMs, but this would be expected to decline over time as there were proportionally more secure vehicles, leaving fewer easy targets.

Subsequent research suggested the introduction of eIMs from 1992 was several years too late to account for the first few years of the theft decline, and suggested that eIMs accounted for just half of the theft decline (Home Office 2015, Morgan and Truman 2017). However this was possibility was ruled out by work showing eIMs were introduced from the mid-1980s and that widespread introduction to high-risk vehicles occurred from 1988 (Farrell and Brown 2015, Farrell 2016, Birks et al. 2016).

#### The Netherlands

The study of the Netherlands by van Ours and Vollaard (2016) can be taken to represent the western European mainland experience of car theft, as indicated by similar cross-national trends (Aebi 2004; van Dijk et al. 2005; Dijk et al. 2005). It was preceded and informed by a German study that developed indicators similar to those described above for the UK (Bässman 2011), and the Dutch study claimed to overcome the methodological limitations of these earlier studies. Its legislative reference point was European Union directive 95/56 which came into force in 1998 and required eIMs on new vehicles across the EU. The study’s design was “based on comparing the theft rates of the same car models with and without the immobiliser as standard option” (van Ours and Vollaard 2016; p. 1266) while accounting for theft risk related to vehicle age. The mandatory introduction of immobilizers provided a ‘natural experiment’ (p. 1268), and the study’s quasi-experimental design with matched control groups is a rigorous design for retrospective evaluation.

The vehicle theft rate in the Netherlands peaked in 1995, fell steeply at first, and had declined 85 percent by 2008 (van Ours and Vollaard 2016; p. 1268). The study concluded that at least 60 percent of the fall in Dutch vehicle theft was due to immobilizers, discounted to 40 percent after considering displacement, noting that “our estimate of the effect of the electronic engine immobilizer provides *a lower bound of the true effect*” (van Ours and Vollaard 2016; p. 1274, emphasis added). The italicized emphasis was added to that quotation because it is likely to be a significant under-estimate, for the following reasons. eIMs were introduced in the Netherlands from 1990, and by 1995 (when the study period began), around one in five vehicles had a factory-installed immobilizer (van Ours and Vollaard 2016; p. 1269). These earlier immobilizers would induce a hot product effect, that is, a disproportionate impact on theft with the riskiest vehicles receiving eIMs. However, this was not captured by the study. This would account for the deceleration in the national theft rate before 1995 and its precipitous decline over the next few years. Note that, had immobilizers only been installed from 1995 onwards then the 1995 peak in Dutch vehicle theft is too early, its turning point too rapid, and the subsequent decline too steep, relative to the proportion of new secure vehicles in the national fleet. The study also excluded sports cars and luxury cars which had high theft rates, reporting that “[i]n 1998 only some 5% of all stolen cars were luxury or sports cars” (p.1272). These high-theft-rate market segments would have received eIMs before 1998 which means that if the iEMs reduced theft by 80% (see below for US estimates) then, other things equal, sports and luxury vehicles alone could previously have accounted for 25% of thefts.^3^

Displacement effects were estimated to reduce eIM impact by a third in the Dutch study. However, only displacement for eIMs fitted to vehicles between 1995 and 1998 was examined. Those are years when displacement rates would be high because many insecure older vehicles remained in circulation, as observed in the UK by Brown and Thomas (2003) and Brown (2004). As the proportion of non-eIM vehicles declined, offender search time and effort would increase and displacement decline accordingly. The long-term displacement rate—the one that matters most—would be minimal because, as van Ours and Vollaard note, “[t]wenty years after the introduction of the engine immobilizer … almost 90% [of all cars on the road] in the Netherlands” had devices installed (p.1268), making it difficult for offenders to find easy alternates.^4^ Overall, then, the Dutch study was appropriate in framing its 60 percent effectiveness estimates as ‘lower bounds’ and a more accurate first approximation of the true effect would encompass all of the theft decline.

#### Australia

Later than its European counterparts, Australian legislation requiring eIM installation on new vehicles came into force in 2001. Australian car theft also peaked in 2001 and declined thereafter. As with the Netherlands and the UK, if eIMs were only introduced from 2001 they would be too late to trigger a national decline in the same year. However, also as elsewhere, there were earlier eIM installation efforts in anticipation of the legislation. The proportion of vehicles with an Australian standard eIM was observed to be 27.4% in 2000 and 64.7% by 2004 (Kriven and Ziersch 2007; p. 115) with the result that “between 2001 and 2014, vehicle theft in Australia fell 65%” (Hodgkinson et al. 2020; p. 99).

Contrary to the findings of the British studies, theft of older vehicles declined at the same time as theft of new vehicles in Australia. This is noteworthy because, if eIMs were fitted only to new vehicles, theft of older vehicles ought not to also decline. It led one study to suggest eIMs only accounted for around half of the car theft decline (Weatherburn and Rahman 2021). However, elsewhere it was observed that Australia had “an older vehicle fleet than in the U.K.” and theft was undertaken by “opportunistic thieves [who] tend to target relatively old vehicles,” which meant that “…no doubt the increased fitting of after-market immobilizers to older vehicles across Australia has made a significant contribution to the overall decline in motor vehicle theft…” (Kriven and Ziersch 2007; pp. 120–121). The decline in theft of older vehicles is explained by Australia’s national multi-year program to retrofit eIMs to older vehicles. That program is documented in the series of annual reports of the Australian National Motor Vehicle Theft Reduction Council (NMVTRC) in the early 2000s^5^ (NMVTRC 2000, 2001, 2002, 2003, 2004, 2005, 2006).

#### Canada

Canadian legislation mandated eIMs on new vehicles from 2007 onwards. As elsewhere, car theft had begun to decline beforehand. Winnipeg, for instance, long known as Canada’s car theft capital, introduced eIMs several years earlier (Linden and Munn-Venn 2008; Linden and Chaturvedi 2005), one study observing that “[a]uto theft in Canada started to fall before national legislation in 2007, likely reflecting earlier adoption encouraged in key cities such as Winnipeg” (Farrell and Brantingham 2013; p. 575). This is consistent with the interpretation of trends in Europe and Australia where anticipatory fitting of eIMs brought down theft earlier than if they were introduced when the legislation came into force.

Between 2003 and 2013, recorded car theft in Canada declined 57% (Boyce et al. 2015) and leveled out across the 2010s. A study of Vancouver found changing spatial patterns of auto theft consistent with vehicle security improvements (Hodgkinson et al. 2016). While a more comprehensive assessment of the Canadian experience would be beneficial, the available evidence is consistent with the introduction and spread of eIMs causing the national car theft decline.

### Discussion

The international evidence is consistent with eIMs being largely, more likely entirely, responsible for the prolonged car theft declines in other high income countries. The dates of eIM introduction varied between countries but each time fitted with the national theft decline. Typically, some eIMs were installed in anticipation of legislative mandates, consistent with an anticipatory benefit effect. This explains why car theft declines sometimes appeared ‘too soon’ to be attributable to legislation.

### Evolution of vehicle security

The invention of the automobile with internal combustion engine is normally dated to 1879 (Gordon 2016, 131). By the first decade of the twentieth century, at least 11 ‘anti joy ride devices’ were used including early ignition circuit breakers with removable plugs, steering wheel locks, speed-limiting devices, and combination locks for hoods (Perry 1910). The National Motor Vehicle Theft Act of 1919 (the Dyer Act) made it a federal offense to transport vehicles across state lines, but there were no other major federal developments until NHTSA was established in the mid-1960 (Maxfield and Clarke, 2009; Lemov 2015). Anti-theft measures that emerged in the first half of the twentieth century included ignition keys, license plates, and registration systems (Karmen 1981; Southall and Ekblom 1985; Newman 2004; Heitmann and Morales 2014).

The 1967 President’s Commission report noted the important of ignition lock quality, given that.

“…Even in those cars taken when the ignition was locked, at least 20 percent were stolen simply by shorting the ignition with such simple devices as paper clips or tin foil. In one city, the elimination of the unlocked ‘off’ position on the 1965 Chevrolet resulted in 50 percent fewer of those models being stolen in 1965 than were stolen in1964.”

(President’s Commission 1967; p. vii)

Following this, the 1968 Motor Vehicle Safety Standard No. 114 required new vehicles to be fitted with improved door locks, buzzers to alert drivers who left the key in the ignition, and ignition locks. Ignition locks prevent a vehicle from being steered or driven forward when the ignition key is removed. An early evaluation found manufacturers met Standard No. 114 by 1969 but recommended minimum performance guidelines for ignition locks because the “effectiveness of specific anti-theft devices … is dependent upon the design ingenuity and the quality of the device” (Barry et al. 1975; p. 17). Further evaluation found the one-third decline in vehicle theft in the 1970s (look back at Fig. 1) was due to the spread of ignition locks which brought vehicle theft “under control for lengthy periods” (Webb 1994; p. 71). While the effect waned as thieves found ways to break ignition locks and hot-wire cars, the finding of effectiveness was corroborated by research on steering wheel locks in Germany and the United Kingdom (Mayhew et al. 1976; Webb 1994; Webb and Laycock 1992; Webb and Brown 2017). This significant national effect of vehicle security was a precursor to the longer-term crime drop that emerged from the 1990s onwards. In the 1980s, though, the crime prevention impact waned as thieves found ways to break ignition locks and hot-wire cars, one study noting that "it is estimated that... perhaps as high as 80 percent of all vehicle thefts are accomplished with one well-know procedure... the thief can work to defeat or bypass the ignition lock. This is typically done by breaking the column cover and forcing the mechanism with a large screwdriver. The technique is known as 'peeling the column' or 'back-driving." (Schroeder and Neuman 1986, 230). 

The politics of the time meant manufacturers were motivated to address car crime. Victim advocates had accused manufacturers of corporate irresponsibility in delaying vehicle security regulation for decades (Karmen 1981, Brill 1982; see Lemov 2015 in relation to vehicle safety). High profile lawsuits against vehicle manufacturers included the 1978 recall of the Ford Pinto and the Ford Motor Company being indicted for the reckless homicide of vehicle occupants (Newman 2004; p. 227; Cullen et al. 1987). It was in this context that what became the 1984 Motor Vehicle Theft Law Enforcement Act was introduced to Congress in 1978, and reintroduced each year until passed (Maxfield and Clarke 2009).

The 1984 Act is central to the present study. It required manufacturers “to mark 14 component parts of selected high-theft automobile lines with identifying numbers.” (Rhodes and Kling 2003; p. 1). The focus on parts-marking was intended to deter professional theft. In the 1950s, over 90 percent of theft was temporary, that is, for joyriding or transportation, usually committed by juveniles, with the vehicle recovered (Savitz 1959). By the 1970s, however, permanent theft for resale or chopping (dismantling for parts) had increased, indicating increasing involvement of professional and organized crime (Harris and Clarke 1991; Rhodes and Kling 2003). Hence, the 1984 Act was intended to promote detection of stolen parts and to deter professional thieves. It indirectly incentivized manufacturers to introduce other anti-theft devices, Sect. 605 (a) of the Act noting that.

“Any manufacturer may petition the Secretary [of Transportation] for an exemption… for any line or line of passenger motor vehicles which are equipped … with an antitheft device which the Secretary determines is likely to be as effective in reducing and deterring motor vehicle theft as compliance with the requirements of such standard.” (H.R. 6257)

In practice, manufacturers were required to undertake parts-marking *unless* they took other adequate security measures, which were primarily eIMs (Brown 2015). Subsequently, the Federal Anti-Car Theft Act of 1992 extended parts-marking requirements to an additional 50 percent of additional vehicles lines for all manufacturers, an extension of the 1984 Act’s framework (Rhodes and Kling 2003). The 1984 Act was a major piece of legislation, but its formal emphasis on parts marking meant that this aspect became the focus of much of the evaluation effort that followed.

### Previous evaluations of the 1984 motor vehicle theft law enforcement act

Evaluations of the 1984 Act were reported in the 1991, 1992 and 1998 Reports to Congress by NHTSA, studies by Abt Associates for the Department of Justice (Rhodes et al. 1997, 1999; Rhodes and Kling 2003), a NHTSA-commissioned study (Maxfield and Clarke 2009) and related independent research (e.g., Harris and Clarke 1991; Clarke and Harris 1992; Hazelbaker 1997). The 1998 NHTSA Report to Congress, building on the 1991 and 1992 reports, concluded that parts-marking may have had an initial effect which quickly subsided:

“Cars with marked parts had lower theft rates than expected, while those with unmarked parts had higher rates than expected. The effect was as strong as 20 percent when cars were new, but it weakened as they became older and seemed to have vanished by the time they were two years old.” (NHTSA 1998; p. xi)

This was interpreted as.

“consistent with the view that many professional thieves subsequently learned how to obliterate the markings, and found them less of a deterrent.” (p. xi)

The report also noted that, as the cars with marked parts got older.

“the benefit diminished, but still persisted at about 6 percent. However, the latter estimate is within the ‘noise range’ of possible biases in the data and it cannot be attributed to parts marking without considerable doubt.” (NHTSA 1998; p. xi)

The evaluation studies were either equivocal or suggested parts marking had a small effect. Maxfield and Clarke’s (2009) study marked a turning point in concluding that “parts-marking seems to have had little effect on vehicle theft in the United States.” (p. 2) and identifying ten limitations of parts-marking. This included that parts-marking “can have no effect on the most common type of theft (joyriding) or theft for export, which appears to be a growing form of professional theft,” along with the “limited knowledge of parts-marking among law enforcement officers and auto-repair businesses,” and the large amount of resources required to initiate an investigation to identify marked parts (Maxfield and Clarke 2009; p. 2). The study critiqued the methodology of previous evaluations to conclude that their findings were over-stated:

“It is [previously] assumed that if a vehicle has received an exemption from parts-making, then it has an anti-theft device. This produces ambiguous classifications of motor vehicles in two ways:

1. Motor vehicles classified as marked [that is, with parts marking], may also have anti-theft devices. In the Abt analysis [of Rhodes, Johnston and McMullen 1999, Rhodes and Kling 2003], this would simultaneously overstate the effectiveness of parts-marking and understate the effectiveness of anti-theft devices.
2. Motor vehicles classified as not-marked and not-exempt may have anti-theft devices. This would understate the effectiveness of anti-theft devices.

In each case, the measurement problem potentially understates the theft-reduction effect of anti-theft devices.” (Maxfield and Clarke 2009; p. 27).

With respect to the evaluation of eIMs, the same report concludes that.

“Another type of measurement problem lies in the broad range of anti-theft devices, and uncertainty about which devices qualify for a parts-marking exemption.” (Maxfield and Clarke 2009; p. 27).

While these criticisms were aimed at the parts-marking evaluations, they also offer methodological challenges that must be met by the present study. We return to this issue later. 

Maxfield and Clarke noted that international studies suggested eIMs were effective, and Maxfield addressed eIMs in a further US study which included “information on anti-theft device installation … for 1991 or newer model year cars only.” (Fujita and Maxfield 2012; p. 234). This compared the change in theft rates for vehicles with and without anti-theft devices and reported that.

“The impact of anti-theft devices is *still limited because the decline in car theft is greater than the decline in cars without such devices* (in other words, the increase in cars with antitheft devices). Between 1990 and 2007, the car theft rate declined by 49 percent, while the number of cars without anti-theft devices decreased by 31 percent and those without immobilizers declined by 19 percent. This might be due to the slow and uneven spread of anti-theft devices in the US compared to other countries where all new cars are required to be equipped with immobilizers.” (Fujita and Maxfield 2012; p. 234, emphasis added)

That is, the study concluded that eIMs were introduced too late, and to too few vehicles, to account for the immediacy and extent of the decline in auto theft. However, it is shown below that eIMs were introduced from 1986 and were strategically targeted at high-risk vehicles to disproportionately impact car theft. As a result, the present study has very different conclusions.

## Data and method

The analysis that follows uses data on vehicle production and thefts, by make and model, published annually in the Federal Register since 1983 by NHTSA:

“The agency [NHTSA] is required by 49 U.S.C. 33104(b)(4) to periodically obtain, from the most reliable source, accurate and timely theft data and publish the data for review and comment.” (Federal Register 2011; 2599)

The theft rate measure derived from the data uses an annual count of theft of new vehicles stolen that year as the numerator and annual counts of vehicle produced that year as the denominator, for each vehicle model. Specifically,

“All theft rates… are given in thefts per 1,000 cars produced. Each theft rate is for cars of the current model year stolen during that same calendar year.” (NHTSA 1992; 14).

To illustrate, Table 1 shows the top ten highest-risk models for 1985. For 1983 to 2014 inclusive, as used here, the Federal Register listed 361 million vehicles produced, including cars, trucks and multipurpose vehicles (MPVs, minivans, or people–carriers), and 1.14 million thefts of new vehicles. Analysis and interpretation of the NHTSA data was not straightforward. The data were cleaned because the Federal Register lists included some changes and inconsistencies in make and model names and notation over time. In addition, the names or design of some vehicle models changed over time, while some vehicle models were terminated and others introduced. The data has other strengths and limitations that are discussed later. Due to its analytic importance, note that the NHTSA measure is the rate of theft of *new* vehicles while the NCVS and UCR measures are of the theft rate for *all* vehicles.

**Table 1:** Top 10 highest-theft-rate models in 1985

Rank	Manufacturer	Model	Production	Thefts	Theft rate
1	General motors	Pontiac Firebird	86,221	1691	19.6
2	General motors	Chevrolet Camaro	167,309	2691	16.1
3	Mazda	RX-7	58,848	864	14.7
4	General motors	Chevrolet Corvette	37,730	543	14.4
5	General motors	Buick Riviera	63,225	908	14.4
6	General motors	Chevrolet Monte Carlo	113,847	1546	13.6
7	General motors	Buick Regal	120,772	1599	13.2
8	General motors	Pontiac Grand Prix	59,790	728	12.2
9	General motors	Cadillac Eldorado	75,215	865	11.5
10	Toyota	Supra	27,442	285	10.4
		Total	810,399	11,720	

(Source: NHTSA)

The tables and analysis included here, including Table 1, were prepared for the present study. The overall analytic approach is the triangulation of information from different sources and indicators, with the specifics of each detailed further below. The aim was to evaluate the impact upon the national vehicle theft rate trend of the introduction and spread of eIMs. 

## Analysis and findings

### Introduction of electronic engine immobilizers

The Fujita and Maxfield (2012) study analyzed eIM data from 1991. However, eIMs were introduced five years earlier and, as should become clear in what follows, the use of data from a later starting date undermined the capacity of that study. The 1998 NHTSA report to Congress notes that eIMs were introduced during manufacture from 1986 onwards:

“One domestic manufacturer gradually introduced factory-installed antitheft devices as standard equipment in a substantial number of make-models during 1986-94.” (NHTSA 1998; p. A-35).

The ‘one domestic manufacturer’ referred to General Motors (GM), the company which dominated the market: Eight of the ten highest-risk vehicle models of 1985 were manufactured by GM (look back at Table 1), accounting for 90 percent of production and theft in the top ten. In 1985, GM produced over 40 percent of new vehicles and over half of thefts of new vehicles (Table 2).^6^

**Table 2:** Vehicle manufacturers in 1985 (total units produced and stolen)

Manufacturer	Production	% Production	Thefts	% Thefts
General Motors	4,641,326	43.3	26,695	54.1
Ford	2,073,419	19.3	7687	15.6
Chrysler	1,226,134	11.4	4205	8.5
Honda	542,635	5.1	1097	2.2
Other	2,236,665	20.9	9645	19.6
Total	10,720,179	100.0	49,329	100

(Source: NHTSA)

With hindsight, the phrase 'one domestic manufacturer' in the NHTSA quotation above is hugely under-stated in implying a minor event. It would have been more accurate to report that, between 1986 and 1994, eIMs were introduced to nearly all of the highest-risk models accounting for most of the nation's car theft. 

### Early assessments

The eIM that GM introduced from 1986 was named The Personalized Automotive Security System (PASS-key):

“In most of the vehicles, the equipment included a specially designed ignition key. A computer in the vehicle reads an encoded capsule embedded in the key and compares it to a microchip within the computer. The ignition system is shut down if the codes do not match, or it is attempted to ‘hot-wire’ the car.” (NHTSA 1998; p. A-35)

While such technology is now commonplace, at the time it was innovative and largely unknown. The PASS-key was installed at manufacture on the two highest-theft 1985 models: the Pontiac Firebird and the Chevrolet Camaro (Table 1).

The 1991 NHTSA Report to Congress noted that

“based on preliminary MY [model year] 1989 theft data, a new antitheft system that at least one manufacturer has installed in one of its car lines, has reduced the theft rates for that line by up to 70 percent.” (NHTSA 1991; p. x)

Similarly, the 1992 NHTSA report to Congress observed a

‘dramatic success story in theft reduction via antitheft systems is that involving the Pontiac Firebird and the Chevrolet Camaro… [comprising] a 67 percent and 65 percent decrease [in theft] for the Firebird and Camaro, respectively’ (NHTSA 1992; p. 21).

**Fig. 2:**
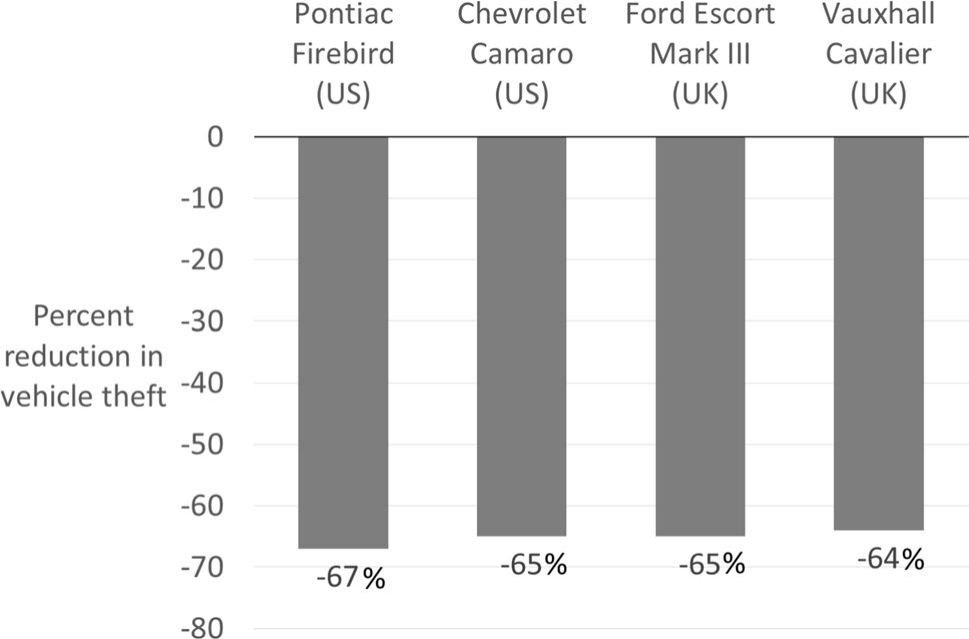
Comparison of evaluations of electronic engine immobilizers (Source: NHTSA 1998, 1992, 2016; Houghton; Farrell and Brown)

These findings are not surprising in the context of the international studies examined above. However, at the time, they were unprecedented. A contemporaneous evaluation by the British government guaged the effect of eIMs on theft of high-risk popular models (Houghton 1992). The similarity between the US and UK findings is uncanny: the first two columns of Fig. 2 represent the two American vehicle models and the second two are the British models.^7^ The two studies, conducted by the responsible government body in different countries, offer corroborating independent evidence.

### A quasi-experimental evaluation

An appendix to the 1998 NHTSA Report to Congress identified the GM vehicle models that received the PASS-key immobilizer from 1986, as well as a matched control group. The vehicle models are shown as Table 3 with rows corresponding to the market types of the vehicle models. For example in the top row, other sporty muscle cars including the Ford Mustang, are controls for the Pontiac Firebird and the Camaro, with the righthand column being.

“control groups of domestic make-models of a similar market class, produced in the same model years that did not get antitheft devices in those years, and did not change their parts-marking status after 1987.” (NHTSA 1998; p. A-36)

**Table 3:** Treatment and control groups

Treatment group	Control group
Camaro, Firebird	Mustang, Daytona, Thunderbird, Cougar
Eldorado, Seville	Lincoln Mark, Continental
DeVille, Riviera, Toronado	Town Car, Mark, Continental
Park Avenue, Oldsmobile 98	Crown Victoria, Grand Marquis
LeSabre, Oldsmobile 88, Bonneville	Taurus, Sable

(Source: NHTSA 1998; A-36)

The park avenue was named the electra prior to 1990

**Fig. 3:**
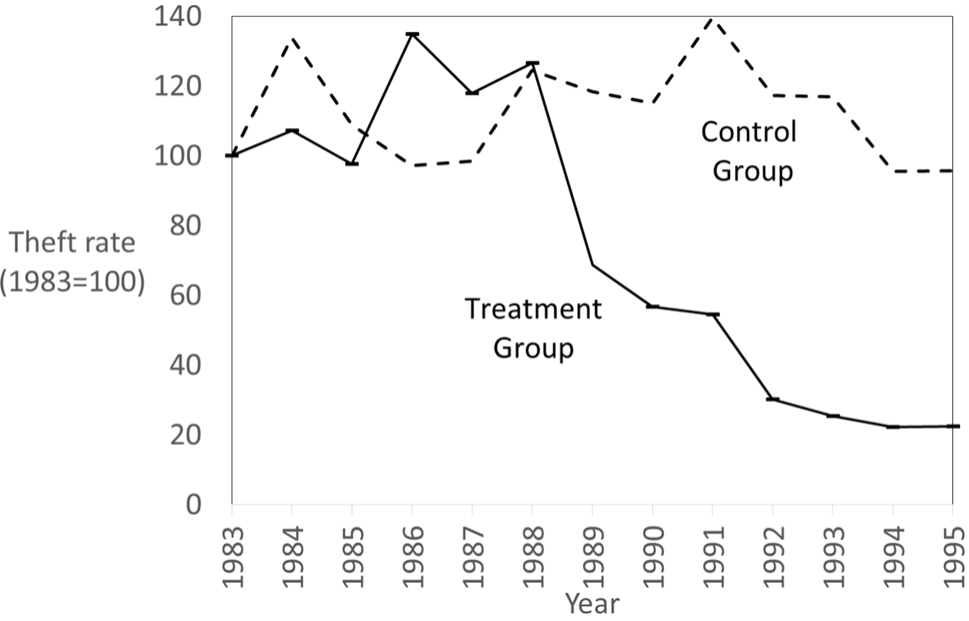
Vehicle theft rates in treatment and control groups 1983–1995 (*n* = 141,385 thefts)

In the NHTSA industry data from the Federal Register, there were 141,385 thefts in the treatment and control groups from 1983 to 1995. The vehicle models listed in Table 3 were combined with their vehicle production and theft data to generate mean annual theft rates for the treatment and control groups. Figure 3 shows the results as indexed theft rate trends based on the rates from Table 4. The control group rate had some natural variation but remained largely unchanged. The pre-intervention theft rate in the treatment group was higher than the control group (reflecting GM’s dominance of vehicle theft), but the mean theft rate of the matched control group was well above the national average. The cliff-edge effect on the treatment group leaves little room for doubt, and from 1992 to 1995, the treatment group theft rate was half that of the control group despite having been higher from 1983 to 1988. Theft in the treatment group declined 78 percent overall, and 77.2 percent relative to the matched control group between these periods, that is, almost 80 percent in both absolute and relative terms.

**Table 4:** Mean theft rates for treatment and control groups

Year(s)	Treatment (T)	Control (C)	% difference (T/C)
1983	8.36	4.04	206.9
1984	8.96	5.41	165.7
1985	8.16	4.40	185.7
1986	11.27	3.93	287.0
1987	9.86	3.98	247.6
1988	10.59	5.03	210.5
1989	5.74	4.79	120.0
1990	4.74	4.65	101.9
1991	4.56	5.64	80.8
1992	2.53	4.74	53.4
1993	2.12	4.72	44.9
1994	1.85	3.86	48.0
1995	1.88	3.87	48.5
t1: Annual average 1983 to 88	9.53	4.46	213.6
t2: Annual average 1992 to 95	2.09	4.30	48.7
% change t1 to t2	78.0%	3.7%	77.2

Here we return to Maxfield and Clarke’s (2009) methodological criticisms of previous evaluation designs. Their first criticism was that previous evaluations had conflated a ‘broad range of anti-theft devices’. This criticism does not apply here because this analysis considers only the eIM. Their second criticism was that previous evaluations conflated the effects of parts-marking with those of eIMs. This does not apply here because, as noted above, the matched control group “did not change their parts-marking status after 1987” (NHTSA 1998; A-36).

### Explanation for the gradual theft decline

**Fig. 4:**
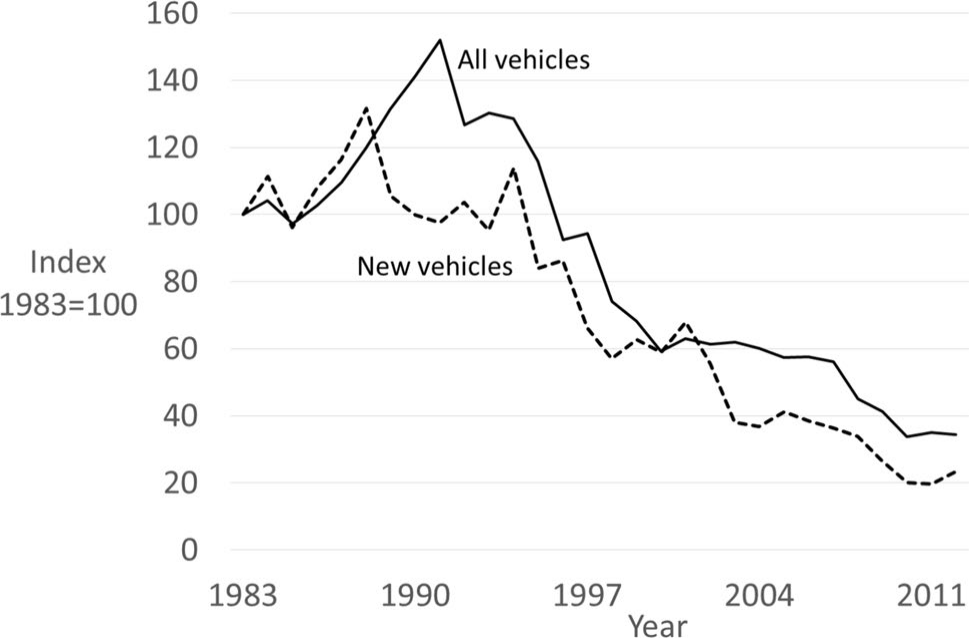
Theft trends for new vehicles and all vehicles 1983–2012 (Source: NHTSA and NCVS)

Figure 4 shows theft rate trends for new vehicles, using the NHTSA data, and for all vehicles, using NCVS data. Four features are of note. First, as engine immobilizers were fitted to new vehicles, theft of new vehicles peaked and fell three years earlier than all vehicles. Second, the theft trends of ‘new’ and ‘all’ vehicles converged somewhat over time, which would be expected as secure vehicles became more prevalent in the national fleet, with full replacement estimated to take 10 years (Laycock 2004; p. 37) to “every fifteen years or so” (Clarke & Harris 1992; p. 3).Third, the theft decline occurred gradually: the annual mean decline from1991 to 2019 was 6.1 percent, consistent with both the gradual spread of immobilizers and the prolonged arms race between offender adaptations and improvements to eIMs (detailed further below). Fourth, the variation in the theft rate as it declined, including some years when the rate increased, is also consistent with the to-and-fro of that arms race.

### Spread of immobilizers

**Fig. 5:**
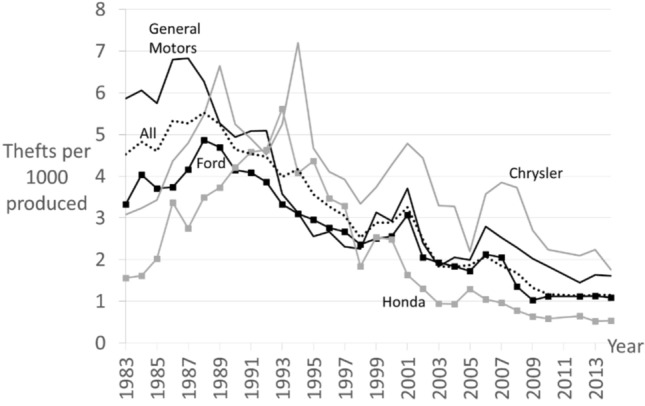
Vehicle theft rates by manufacturer 1983–2014 (Source: NHTSA)

Mean theft rates for the main manufacturers’ vehicle fleets are shown in Fig. 5 using the NHTSA production and theft data. Recall that, in 1985, GM accounted for 41 percent of production and 54 percent of thefts (Table 2). Following GM’s introduction of the immobilizer from 1986, the 1992 NHTSA Report to Congress noted that GM “claims that by MY [model year] 1994 the majority of GM cars, approximately 2.6 million, are scheduled to have some version of the PASS-KEY system as standard equipment.” (NHTSA 1992; 25). This means that remaining models (without immobilizers) were low-risk. By 1995, when immobilizers were widespread on its high-risk models, GM accounted for 31 percent of production but it share of thefts of new vehicles was had fallen to 22 percent.

A Ford immobilizer was documented in the American-UK comparison examined earlier (Houghton 1992), and Ford’s theft rate fell from 1989. As GM and Ford protected their high-risk vehicles, the theft rates of Chryslers and Hondas increased, likely as partial displacement to next-best similar easy targets in the early years, with displacement declining over time as fewer easy targets were available. Chrysler’s theft rate peaked in 1994 by which time five of the top-ten highest risk, and a quarter of new vehicles stolen, were Chryslers (the highest-risk Chryslers at that time being the Plymouth Sundance and the Lebaron Sedan). The lower theft rate of Hondas likely reflected thieves’ preference for domestic muscle cars plus the relative difficulty of stealing Japanese models with their ‘awkward locks’ noted by a juvenile thief:

“I just look for cars that are easy to [steal]—cars that aren’t alarmed, general stuff like Fords … that are easy to get into. …[whereas] Japanese makes …are really hard to get into because they’ve got awkward locks.” (offender quoted in Light, Nee & Ingham 1993, pp.48–9)

Low theft rates of Hondas meant there was no formal requirement to improve security. This changed as theft rates rose in the early 1990s, also likely due to partial displacement as other manufacturers’ vehicles became harder to steal (see also Barro 2014). The gradual permeation of the market by eIMs is evident in a subsequent NHTSA report’s observation that.

“Ford Motor Company claimed that its MY 1997 Mustang vehicle line (with an immobilizer) led to a 70 percent reduction in theft compared to its MY 1995 Mustang (without an immobilizer). Chrysler Corporation informed the agency that the inclusion of an immobilizer device as standard equipment on the MY 1999 Jeep Grand Cherokee resulted in a 52 percent net average reduction in vehicle thefts… Mitsubishi Motors Corporation informed the agency that the theft rate for its MY 2000 Eclipse vehicle line (with an immobilizer device) was almost 42 percent lower than that of its MY 1999 Eclipse (without a immobilizer device). Mazda Motor Corporation reported that a comparison of theft loss data showed an average theft reduction of approximately 50 percent after an immobilizer device was installed as standard equipment in a vehicle line.” (NHTSA 1991; p. 66835, footnotes removed)

While GM was the market leader that cooperated with NHTSA and first introduced the eIM, the subsequent cascade effect, as immobilizers were more widely introduced, is consistent with the diffusion of technology in a competitive market (Rogers 1962).

### The prolonged co-evolutionary arms race

Crime prevention sometimes provokes offender adaptations which, in turn, prompts further crime prevention development, and so on. This iterative process has been described as a co-evolutionary arms race (Ekblom 1999; Brown 2017). In relation to vehicle theft, offender adaptations were evident in the development of immobilizer by-pass technologies, car key-theft burglaries, the targeting of higher-end vehicles for resale and export markets and, more recently, the hacking of on-board computers (Clarke and Brown 2003, Donkin and Wellsmith 2006, Copes and Cherbonneau 2006, Barro 2014, Brown 2017; Hodgkinson et al. 2020, Vellinga 2022, Polanco and Cheng 2022, Jacobson 2023).

A series of manufacturer responses are also well documented. The first PASS-key immobilizer was replaced by the PASS-key II immobilizer following the identification of weaknesses, anassessment by the Highway Loss Data Institute (HLDI) finding that.

“Comparing the 1994 GM passenger cars equipped with this device and the 1993 counterpart models without the device (no other design changes), HLDI found that the average loss payment declined dramatically for vehicles with PASS-Key II. … According to a new study of 1995 BMWs, average loss payments dropped significantly when a passive immobilizing antitheft device became standard in midyear…” (Hazelbaker 1997; p. 289).

The Pass-key II was superseded in the late 1990’s by the Passlock which was designed to be tamper-proof. It proved otherwise and was followed by transponder systems requiring the presence of the key to transmit a signal allowing the engine to be started, which was followed by Powerlock (which disabled the starter system) and biometric systems (Maxfield and Clarke 2009; p. 162–163) and, more recently, cyber-security responses to hacking (Polanco and Cheng 2022). There is also international evidence of continued eIM improvements. In the UK, six generations of immobilizer were institutionalized via a series of industry standards issued under the new vehicle security assessment (NVSA) program between 1993 and 2014 (Briggs 2014). 

The co-evolutionary arms race has continued. In the 2020’s, when a security flaw in Hyundai and Kia vehicles produced a theft spike, the response was a software update aiming to closed-off the crime opportunity (Economist 2023; Purdy 2023). Overall, then, a series of offender adaptations has been countered by security improvements. Of course, continued success is not guaranteed and this suggests manufacturers should continually invest in security research and development.

## Discussion

The twentieth century was 'the American century' (Evans 1998). The domestic automobile industry was a key components of US global economic, political and cultural success, one economic historian concluding that "[f]ew inventions in human history have equalled the internal combustion engine in their effects" (Gordon 2016, 149).                                                                                                                                                                                                   In this study, a re-assessment of the international literature found eIMs responsible for prolonged crime declines in Australia, Britain, Canada, Germany and the Netherlands, the latter effectively representing continental western Europe as a whole. Central to the re-assessment was the identification of how eIMs were typically introduced before legislation came into force. Consistent with the concept of anticipatory benefits (Smith et al. 2002), this meant theft rates declined before, or coincident with, legislation. There was already consensus that eIMs reduced vehicle theft, but the finding they account for the whole of each prolonged car theft decline considerably furthers our understanding.

The 1984 Motor Vehicle Theft and Law Enforcement Act provided a regulatory incentive for US manufacturers to improve security on high-risk vehicles, inducing what has subsequently become known as a hot-products strategy. General Motors was the dominant manufacturer and introduced eIMs to most of the nation's highest-risk vehicles between 1986 and 1994. This had a dramatic effect, documented here with evidence from contemporaneous congressional reports and data. A cliff-edge 80 percent reduction in theft was demonstrated in a reconstructued quasi-experimental evaluation using industry data from the Federal Register, and  the installation of eIMs cascaded through the car industry in the years that followed. The theft rate fell earlier for new vehicles, consistent with new vehicle having reduced risk due to security improvements. The national theft rate declined gradually as new secure measures spread. The prolonged and variable rate of decline reflected the well documented arms race between thieves and manufacturers. Overall, the evidence shows an effective situational crime prevention intervention underpinned by the mechanisms of crime opportunity theory and a hot-products strategy, consistent with the security hypothesis explaining the prolonged drops in many crimes in the US and elsewhere.

### Study limitations

Further examination of the contribution of other security technologies, particularly door deadlocks, would be a useful contribution if relevant data can be identified. The quasi-experimental evaluation of eIMs used non-identical matched comparison groups, but the cliff-edge treatment effect with close to an 80 percent reduction in theft, in both absolute and relative terms, leaves no real room for doubt, and squares with findings from elsewhere.

Critics will likely claim that efforts to circumvent immobilizers make their long-term sustainability uncertain. This is a separate issue from the causes of the long-term decline. However, it strengthens the case for continual security improvements by manufacturers. Critics may also claim that car theft fell for other reasons. Such critics would first need to explain how any other explanation might account for the cliff-edge decline in theft of the treatment but not control group, the earlier decline in new vehicle theft, and other such evidence.

### Broader implications

The 1967 US President’s Commission noted that

“…[m]any crimes would not be committed, indeed many criminal careers would not begin, if there were fewer opportunities for crime. … Auto theft is a good example…. Another major reason that it is important to reduce auto theft is that stealing a car is very often the criminal act that starts a boy [or other young person] on a course of lawbreaking.” (1967; p. vii).

This effect is elsewhere identified as a debut crime effect that works through offender age cohorts over time (Farrell et al. 2015; Dixon and Farrell 2020). Debut crime are the easy crimes from which adolescents learn and, if rewarding, progress. If the eIM stemmed adolescent involvement and continuance in offending and induced the decline in other crimes including violence, this would mean the eIM was central to the international crime drop more generally. 

The findings suggest there are evidential grounds to reconsider theories of criminal behaviour: criminality is a more marginal and less pathological activity than often considered, and largely dependent on how easy it is to commit crime.

With respect to policy and practice, consideration should be given to legislation that requires factory-installed eIMs for all new US vehicles (with eIM standards to ensure their continual improvement). More broadly, businesses have little interest in preventing crime opportunities produced by their products and services, because the crime costs are paid by victims and society. To reduce these crime externalities, government should introduce incentives, disincentives and regulation similar to those used for controlling other forms of pollution. This is necessary because businesses have the technical expertise to reduce these crime opportunities whereas police and other agents (including academics) do not. The efficient way to address crime problems is to focus on crime concentrations including but not limited to hot products, repeat victimization, risky facilities, risky transport routes, and geographic hot spots.  

## Conclusion

The vehicle electronic engine immobilizer caused the 80 percent decline in vehicle theft in the United States. Consideration should be given to legislation requiring factory-installed eIMs for all new vehicles. A critical re-assessment of the international literature concluded that eIMs account for the prolonged car crime declines internationally. The study findings are consistent with the theoretical frameworks of crime opportunity theory, particularly the hot products strategy, situational crime prevention, anticipatory benefits and the limited effects of crime displacement. If the decline in vehicle theft induced the prolonged declines in other crime types including violence, the electronic engine immobilizer may be the most important crime prevention device of recent history.

## Data Availability

The industry data used in the analysis is publicly available in the Federal Register.

## Footnotes

1 Number of vehicles in operation and total households from Oakridge National Laboratory’s *Transportation Energy Data Book*, Table 9.01 at https://tedb.ornl.gov/data/ accessed 19 October 2022. Figure 1 uses published NCVS rates per 1,000 households converted to rates per 1,000 vehicles in operation.

2 Opening statement of Chairman Charles E. Schumer, Chair of the House of Representatives Subcommittee meeting to consider evidence relating to the Anti-Car Theft Act 1992, Monday 9 December 1991; H.R. 4542, 1991.

3 The figure changes with the estimate of effectiveness but the principle remains the same.

4 The possibility of offender adaptations to circumvent immobilizers are discussed further below.

5 All NMVTRC annual reports are available at https://carsafe.com.au/publications.

6 Manufacturers are referred to here following the convention of Table 2. For present purposes, Chrysler includes Daimler (DaimlerChrysler existed from 1998 to 2007) and Honda includes Acura.

7 Four model were included in the British evaluation and the two shown had the largest effects. The two not shown used early-version immobilizers that reduced theft by around a third.
